# Soybean (*Glycine max*) INFOGEST Colonic Digests Attenuated Inflammatory Responses Based on Protein Profiles of Different Varieties

**DOI:** 10.3390/ijms241512396

**Published:** 2023-08-03

**Authors:** Jennifer Kusumah, Erick Damian Castañeda-Reyes, Neal A. Bringe, Elvira Gonzalez de Mejia

**Affiliations:** 1 228 Edward R Madigan Lab, Department Food Science, and Human Nutrition, University of Illinois at Urbana-Champaign, Urbana, IL 61801, USA; kusumah2@illinois.edu (J.K.); edreyes@illinois.edu (E.D.C.-R.); 2 Benson Hill Company, St. Louis, MO 63132, USA; nealb@truevine.net

**Keywords:** colonic digestion, COX-2, soybean (*Glycine max*), inflammation, 5-LOX, iNOS, NF-κB

## Abstract

Soybean compounds have been established to modulate inflammation, but less is known about how whole soybean compositions work together after digestion. The objective was to evaluate and compare the anti-inflammatory responses of different soybean varieties under simulated gastrointestinal digestion, with additional consideration of the glycinin:β-conglycinin ratio (GBR). Soybean colonic digests (SCD) inhibited cyclooxygenase (COX)-2 (25–82%), 5-lipoxidase (LOX) (18–35%), and inducible nitric oxide (iNOS) (8–61%). Varieties 88, GN3, and 93 were the most effective inhibitors. SCD (1 mg/mL) of varieties 81 and GN1 significantly (*p* < 0.05) reduced nitrite production by 44 and 47%, respectively, compared to lipopolysaccharide (LPS)-stimulated macrophages. SCD effectively reduced pro-inflammatory cytokine interleukin (IL)-6 (50 and 80% for 96 and GN1, respectively). Western blot results showed a decrease in the expression of iNOS, p65, and p50. The GBR was in the range of 0.05–1.57. Higher ratio correlated with higher production of IL-1β (r = 0.44) and tumor necrosis factor-alpha (TNF-α, r = 0.56). Inflammatory microarray results showed a significant decrease in expression of markers granulocyte-macrophage colony-stimulating factor (GM-CSF) and IL-6 in cells treated with GN1 SCD compared to LPS. The results suggested that SCD exerted its anti-inflammatory potential through nuclear factor kappa B (NF-κΒ) pathway inhibition by decreasing the levels of NF-κB-dependent cytokines and subunits, and inhibition of pro-inflammatory enzyme activity.

## 1. Introduction

Soybean is a legume initially cultivated in China, and its consumption has been widespread in many Asian countries [1]. The United States is one of the world’s leading producers of soybean. The incorporation of soybean into the Western diet has been gaining more interest due to evidence of its health benefits [2]. The main storage proteins of soybean are glycinin and β-conglycinin, making up 70% of the total seed protein [3]. The proportion of glycinin and β-conglycinin in different soybean varieties influences peptide release and antioxidant capacity under simulated gastrointestinal digestion conditions [4].

Further digestion of soybean, continuing from the gastrointestinal to colonic phase, may be able to release more bioactive peptides that have health benefits; there is potential for dietary peptides to be absorbed in the human colon [5]. However, more studies are needed on colonic digestion and absorption of proteins and peptides.

The anti-inflammatory properties of soybean have been demonstrated by previous studies [6,7,8]. Inflammation is part of the defense mechanism of the body, triggered by harmful stimuli such as microbial infection or damage signals. The inflammation over-activation leads to major affliction over time [9]. Chronic inflammation has been identified as part of the progression of many chronic diseases and thus has received much attention in research [10]. Three different classical signaling pathways have been identified for inflammation: the Janus kinase/signal transducer and activator of transcription (JAK/STAT), the mitogen-activated protein kinase (MAPK), and the nuclear factor kappa-B (NF-κB) [11]. In addition to those pathways, enzymes such as cyclooxygenase-2 (COX-2), lipoxygenase (LOX), and inducible nitric oxide synthase (iNOS) are associated with inflammation. Similarly, inhibiting these enzymes has also been the basis of developing anti-inflammatory drugs such as aspirin [12]. COX-2 is involved in inflammation by converting arachidonic acid into prostaglandins [13]. Similarly, LOX is also involved in converting arachidonic acid into pro-inflammatory leukotrienes [14].

In vitro studies using macrophages challenged with lipopolysaccharides (LPS) revealed that soybean bioactive compounds target the NF-κB pathway, inhibiting the phosphorylation of the p65 subunit and the IκB kinase complex (IKK)-α/β and decreasing pro-inflammatory biomarkers such as tumor necrosis factor (TNF)-α, nitric oxide (NO), iNOS, prostaglandin E_2_ (PGE_2_), and interleukin (IL)-6 [15]. Additionally, soybean bioactive compounds also inhibit the enzymatic activity of COX-2 and LOX [16,17].

We hypothesized that soybean varieties having different protein profiles and concentrations would differ in their anti-inflammatory activity. The objective was to evaluate and compare the anti-inflammatory responses of soybean variety digests on the inflammatory signaling pathways in vitro and biochemically, and particularly, how the proportion of glycinin and β-conglycinin would affect the response. This study focused on the NF-κB pathway of inflammation and specific biomarkers p50, p65, NO, IL-1β, IL-6, and TNF-α. This study was unique as it was the first to explore the anti-inflammatory effects of soybean varieties with different protein concentrations and profiles under simulated colonic digestion.

## 2. Results

### 2.1. Degree of Hydrolysis and Protein Profile from Sodium Dodecyl-Sulfate Polyacrylamide Gel Electrophoresis (SDS–PAGE) Analysis

**Figure 1:**
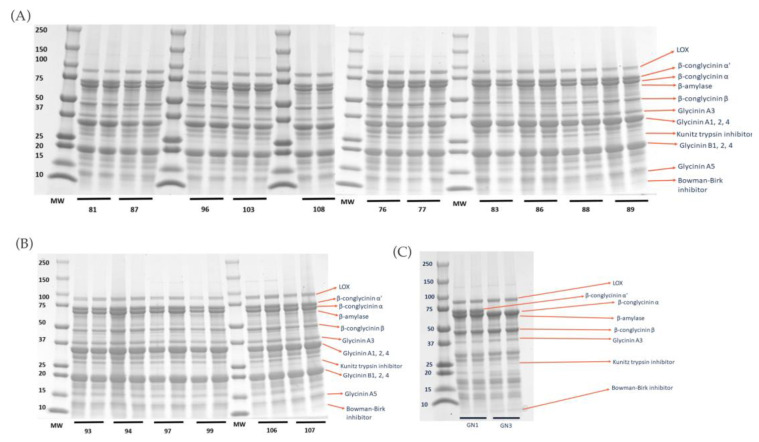
SDS–PAGE results of extracted raw protein from seventeen soybean varieties and two additional glycinin-null varieties. (**A**) Varieties 81, 87, 96, 103, 108, 76, 77, 83, 86, 88, and 89. (**B**) Varieties 93, 94, 97, 99, 106, and 107. (**C**) Glycinin-null varieties GN1 and GN3. Relevant protein bands have been identified according to their molecular weight: enzymes lipoxygenase (LOX) and α-amylase, acidic and basic subunits of glycinin and β-conglycinin, and anti-nutritional factors Kunitz trypsin inhibitors (KTI) and Bowman–Birk inhibitors (BBI).

Analysis of SDS–PAGE results (Figure 1) of all the extracted raw proteins from all varieties revealed protein profiles, including enzymes such as LOX and β-amylase, basic and acidic subunits of glycinin, β-conglycinin α, α’, and β, and protease inhibitors such as Kunitz trypsin inhibitor (KTI) and Bowman–Birk inhibitor (BBI). Supplementary Table S1 presents the identification of proteins in soybean varieties and their respective approximate molecular weight ranges.

**Figure 2:**
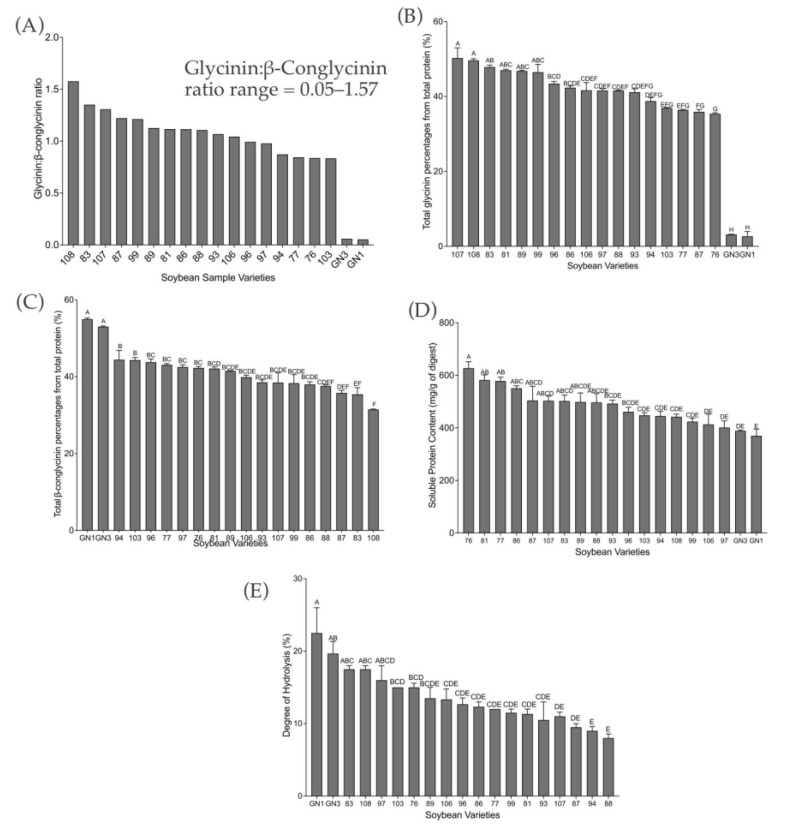
(**A**) Glycinin:β-conglycinin ratio for each soybean variety. (**B**) The percentage of total glycinin and (**C**) β-conglycinin of the total protein content. (**D**) Soluble protein mg/g of soybean colonic digests. (**E**) Degree of hydrolysis expressed as a percentage. The assay was performed in triplicate, and an average was taken. Bars with different letters represent statistically significant differences according to one-way ANOVA and Tukey’s multiple comparison tests (*p* < 0.05). All results were arranged from highest to lowest.

The glycinin:β-conglycinin ratio of the varieties (Figure 2A) was in the range of 0.05–1.57. Variety 108 (1.58) had the highest ratio, and variety GN1 (0.05) had the lowest ratio, confirming that the varieties have different protein profiles. Varieties glycinin-null (GN) GN1 and GN3 had lower glycinin content, as expected; however, a marked difference between these two varieties was the absence of β-conglycinin subunit α’ in variety GN3. Variety 107 was found to have one of the highest glycinin content values (50%), although not statistically different from 108, 83, 81, 89, and 99. Variety GN1 (3%) had the lowest (Figure 2B). The variety GN1 was also found to have one of the highest β-conglycinin content values (53%) and variety 108 (32%) one of the lowest (Figure 2C). Soluble protein concentration (Figure 2D) ranged from 369.8 mg/g variety GN1digest to 626.9 mg/g variety 76 digest. Varieties GN1 and GN3 had among the highest degrees of hydrolysis among the varieties tested (Figure 2E).

### 2.2. Peptide Sequencing

Four different peptides unique to the colonic digestion phase were found: EF (Glu-Phe; mass = 294.1 Da, pI = 3.1, net charge = −1, hydrophobicity = 9.8 kcal/mol); EY (Glu-Tyr; mass = 310.1 Da, pI = 3.1, net charge = −1, hydrophobicity = 10.8 kcal/mol); CSR (Cys-Ser-Arg; mass = 364.2 Da, pI = 8.7, net charge = 1, hydrophobicity = 8.7 kcal/mol); AIGIN (Ala-Ile-Gly-Ile-Asn; mass = 486.3 Da, pI = 5.4, net charge = 0, hydrophobicity = 8.2 kcal/mol). The parental proteins for these peptides were 7S globulin for EY and CSR, and β-conglycinin-α for EF and AIGIN.

### 2.3. Biochemical Screening of Pro-Inflammatory Markers

**Figure 3:**
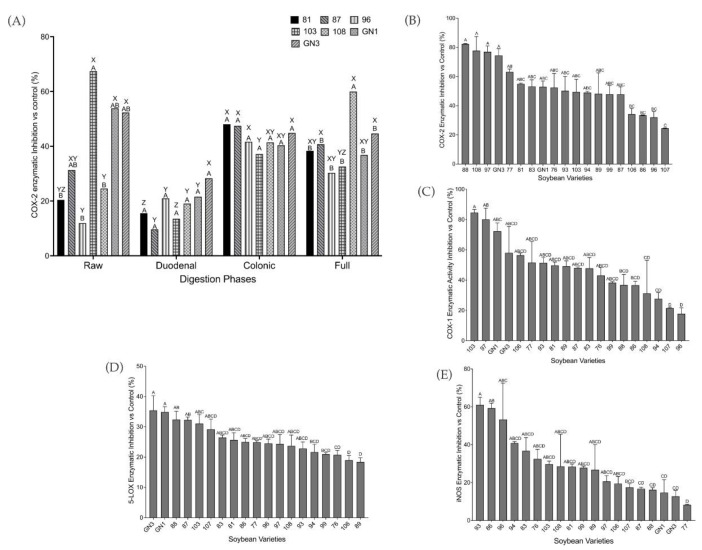
Results of anti-inflammatory inhibitor screening assays. (**A**) COX-2 inhibitor screening assay results for five representative soybean digests and two additional glycinin-null digests were obtained from three different digestion phases: duodenal, colonic, and full, as well as the extracted raw protein from each variety. (**B**) COX-2 and (**C**) COX-1 inhibitor screening assay results of all nineteen different soybean colonic digests expressed as a percentage of the enzyme control without any inhibitor present. (**D**) 5-LOX inhibitor screening assay expressed as a percentage of enzyme inhibition with respect to control. (**E**) iNOS inhibitor screening assay expressed as a percentage of enzyme inhibition with respect to control. Bars show the mean and standard deviation. The blank digest was used as a negative control in the assays (6% COX-1 enzyme inhibition, 15% COX-2 enzyme inhibition, 8% 5-LOX enzyme inhibition, and 6% iNOS enzyme inhibition). SC-560 was used as the positive control for the COX-1 inhibitor screening assay (91% COX-1 enzyme inhibition), and DuP-697 was used as the positive control for the COX-2 inhibitor screening assay (96% COX-2 enzyme inhibition). Zileuton (100 μM) was used as a positive control (90% 5-LOX enzyme inhibition. Diphenyleneiodonium (DPI, 1 mM) was used as a positive control with 91% iNOS enzyme inhibition. Bars with different letters represent statistically significant differences according to one-way ANOVA and Tukey’s multiple comparison tests (*p* < 0.05). Results were arranged from highest to lowest values. Letters A–D represent significant differences among varieties; letters X–Z represent significant differences among digestion phases.

COX-2 was assessed to evaluate the anti-inflammatory properties of the different soybean varieties after simulated digestion. The highest COX-2 inhibition was achieved after colonic and full digestion for most of the varieties (Figure 3A), with up to 60% inhibition by variety 108, followed by colonic digestion, which inhibited COX-2 up to 48% in variety 81. The lowest inhibition was shown by duodenal digestion, up to 29% in variety GN3. No statistically significant difference was found in the COX-2 inhibition by soybean digests from both colonic and full digestions. However, there was a significant difference (*p* < 0.05) in the COX-2 inhibition by soybean digests from duodenal digestion compared to the other two digestion conditions. A statistically significant difference (*p* < 0.05) was also observed between extracted raw protein and digests from both full and colonic digestion phases. Since soybean digests from colonic and full digestion behave similarly, soybean colonic digestion was selected for further analyses. Significant differences (*p* < 0.05) in COX-2 inhibition were observed among varieties for extracted raw protein, and for colonic or full digestion phases.

Comparing the colonic digests of different varieties, the COX-2 (Figure 3B) was inhibited from 25 to 82%, and COX-1 inhibition (Figure 3C) ranged from 18 to 85%. The 5-LOX inhibitory screening assay (Figure 3D) showed promising enzymatic inhibition by soybean colonic digests. The enzymatic inhibition ranged from 18 to 35%, with varieties GN3 and 89 exhibiting the highest and lowest inhibition, respectively. The iNOS inhibition (Figure 3E) showed promising anti-inflammation properties of SCD by inhibiting the iNOS enzymatic activity. The highest inhibition was by variety 93 (61%), and the lowest was by variety 77 (8%).

### 2.4. Correlation Analysis

**Figure 4:**
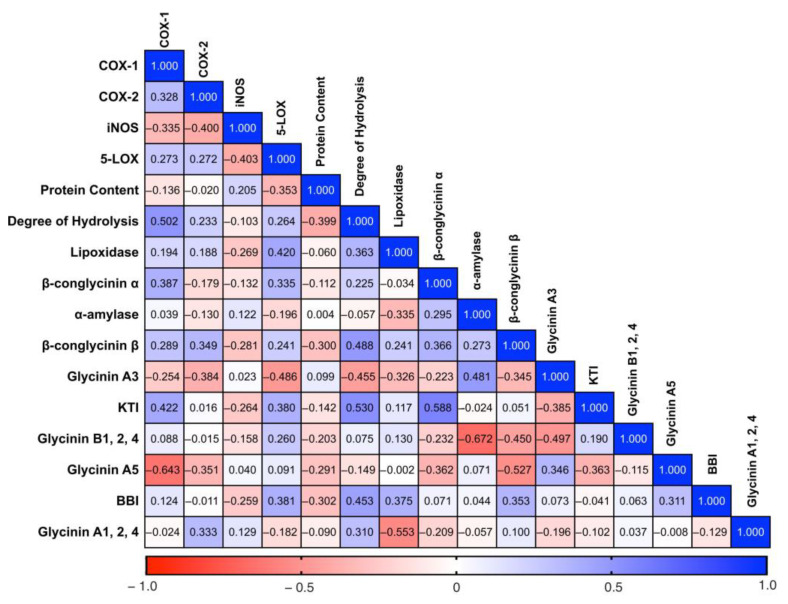
Results of correlation analysis according to Pearson correlation coefficient method performed using GraphPad Prism 9 software. A correlation was considered when r > 0.5 or r < −0.5 and *p* < 0.05. Heatmap of the correlation matrix summarizing the correlation analysis results. COX, cyclooxygenase; iNOS, inducible nitric oxide synthase; LOX, lipoxygenase; KTI, Kunitz trypsin inhibitor; BBI, Bowman–Birk inhibitor.

The correlations (Figure 4) showed that the inhibition of COX-1 was positively correlated with the degree of hydrolysis (r = 0.50, *p* < 0.05) and negatively correlated with glycinin A5 (r = −0.64, *p* < 0.05). The degree of hydrolysis was positively correlated with KTI (r = 0.53, *p* < 0.05). Higher total glycinin content was fairly associated with COX-2 (r = 0.33). Supplementary Figure S1 presents the proportion of each protein expressed as a percentage of the total protein.

### 2.5. Cell Viability and In Vitro Pro-Inflammatory Biomarkers

**Figure 5:**
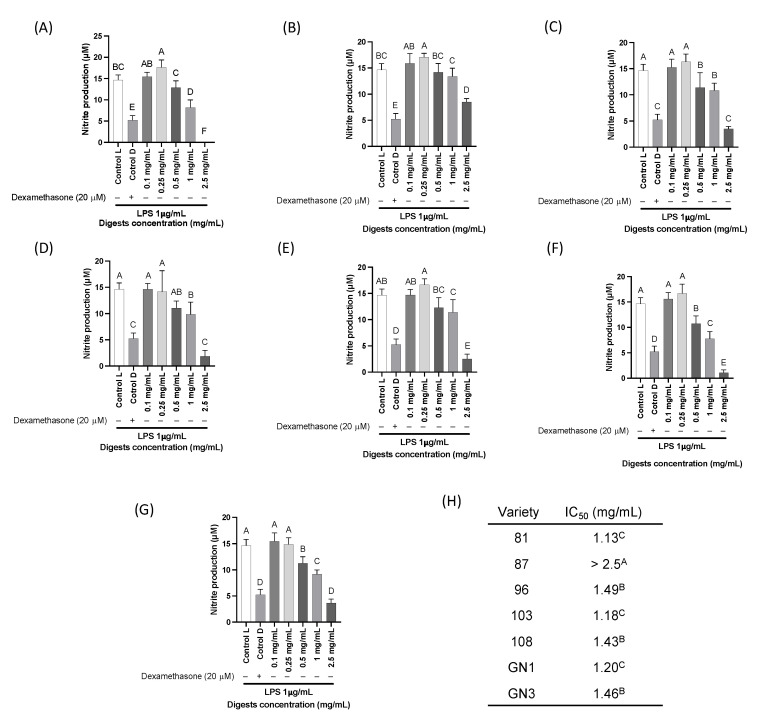
Nitrite production of LPS-stimulated RAW264.7 treated with different soybean digest concentrations (0.1–2.5 mg/mL) of seven varieties 81 (**A**), 87 (**B**), 96 (**C**), 103 (**D**), 108 (**E**), GN1 (**F**), and GN3 (**G**). (**H**) IC_50_ of percentage nitrite production inhibition. The assay was performed at least in duplicate. Dexamethasone (20 μM) was used as a positive control to inhibit nitrite production. Data were analyzed using ANOVA (one-way) and Dunnett’s post hoc test. Bars with different letters represent statistically significant differences at *p* < 0.0001.

Seven different varieties including two glycinin-null varieties were selected to proceed to in vitro analysis. These seven varieties were the ones that consistently exhibited highest inhibition of activity of the three different pro-inflammatory enzymes in biochemical screening. According to the cell viability results (Supplementary Figure S2), the treatments with soybean colonic digests did not yield any cytotoxic effects to the macrophages. In general, the nitrite production (Figure 5) significantly decreased when applied to the different soybean digests at a concentration > 1 mg/mL (*p* < 0.05) in comparison with the LPS-stimulated control. Variety 81 (Figure 5A) was found as the variety with the lowest IC_50_ (1.1 mg/mL). Variety 87 (Figure 5B) was not able to inhibit in 50% the nitrite production at the higher treated concentration (2.5 mg/mL), inhibiting 15% of the nitrite production at 1 mg/mL compared with the LPS-stimulated control. Variety 87 was the least effective in inhibiting nitrite production.

### 2.6. Enzyme-Linked Immunoassays in In Vitro Results

**Figure 6:**
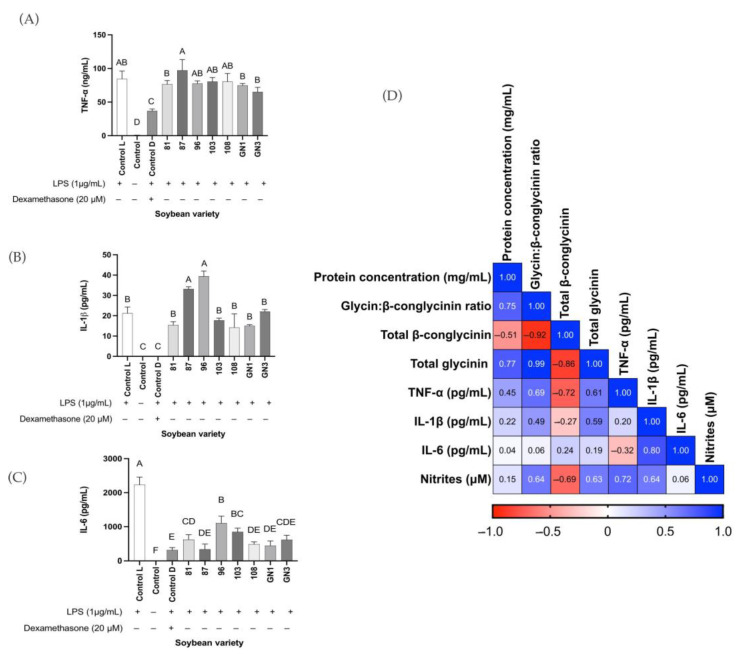
Results from ELISA. (**A**) Tumor necrosis factor (TNF)-α (ng/mL). (**B**) Interleukin (IL)-1β (pg/mL). (**C**) IL-6 (pg/mL). (**D**) Correlation matrix. A correlation was considered when r > 0.5 or r < −0.5 and *p* < 0.05. The cells were stimulated with LPS (1 μg/mL) and treated with the IC_50_ values of nitrite production of selected soybean digests based on the biochemical results. Heatmap of the correlation matrix summarizing the correlation analysis results. The assays were performed at least in duplicate. Data were expressed as the concentration of biomarkers produced (pg/mL or ng/mL) and analyzed using one-way ANOVA and Tukey’s post hoc test. Bars with different letters represent statistically significant differences at *p* < 0.05.

Production of biomarkers TNF-α, IL-1β, and IL-6 in RAW264.7 macrophages stimulated with LPS are presented in Figure 6. TNF-α was not significantly different among varieties tested and the control (*p* > 0.05). In general, production of TNF-α was not significantly reduced after treating the cells with the soybean digests (Figure 6A). IL-1β was not significantly different among varieties tested and the control (*p* > 0.05) except for varieties 87 and 96 (Figure 6B). The production of IL-6 decreased significantly compared with the LPS-stimulated macrophages (*p* < 0.05). Variety GN3 inhibited the production of IL-6 (67%), not different than varieties GN1, 108, and 87 (Figure 6C). Variety 87 inhibited IL-6 up to 85% (Figure 6C), similar to the treatment with dexamethasone, followed by GN1 (80%), 108, and GN3. The correlation results (Figure 6D) showed that the concentration of TNF-α was negatively affected (r = −0.72, *p* < 0.05) by total β-conglycinin content, but it was positively affected by the glycinin:β-conglycinin ratio (r = 0.69, *p* < 0.05). Similarly, nitrite production was negatively affected by the β-conglycinin content (r = −0.69, *p* < 0.05). Protein concentration was not found to have a strong correlation with the production of any of the inflammatory markers tested.

**Figure 7:**
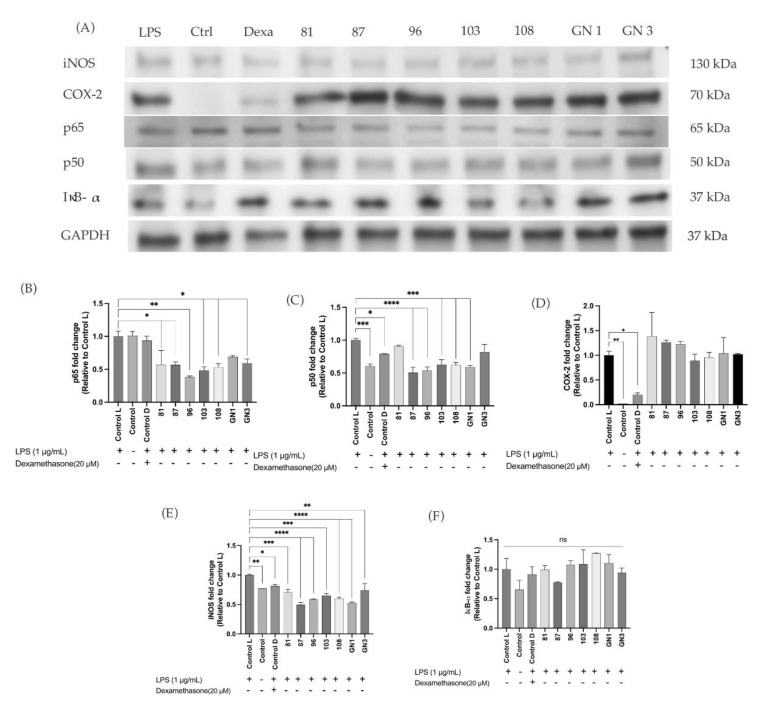
(**A**) Western blot images for intensity analysis including markers iNOS, COX-2, p65, p50, IκB-α, and GAPDH. (**B**) p65 fold-change expression relative to the control after treatments. (**C**) p50 fold-change expression relative to the control after treatments. (**D**) COX-2 fold-change expression relative to the control after treatments. (**E**) iNOS fold-change expression relative to the control after treatments. (**F**) IκB-α fold-change expression relative to the control after treatments. The experiments were performed at least in duplicate. Protein expression was expressed as a percentage compared to the control treated with LPS normalized to either GAPDH and analyzed using one-way ANOVA and Tukey’s post hoc test. Asterisks * represent statistically significant differences at *p* < 0.05, ** at *p* < 0.01, *** at *p* < 0.001, and **** at *p* < 0.0001; ns means no significance. The contrast of western blot images were adjusted for visual purposes (gamma = 0.25 for the p65 result, gamma = 0.5 for the rest of the results.).

Western blot results (Figure 7A) showed that SCD treatments significantly decreased the expression of NF-κB subunits p65 (Figure 7B) and p50 (Figure 7C) compared to control LPS. Results showed no statistical differences in the expression of COX-2 in SCD in comparison to the control L (Figure 7D). There was a significant decrease in expression of pro-inflammatory iNOS with varieties 87, 96, 108 and GN1 (Figure 7E). The expression of IκB-α was not statistically different among treatment (Figure 7F).

**Figure 8:**
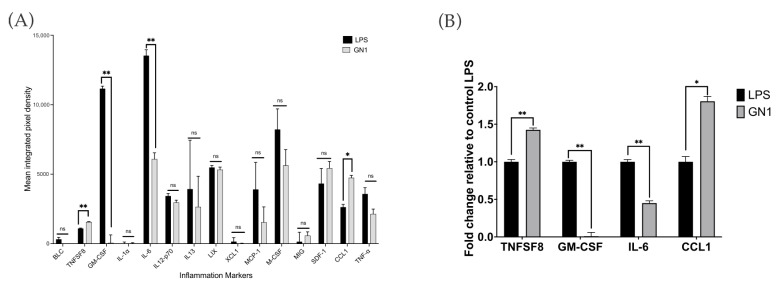
Inflammation antibody array results expressed as (**A**) mean integrated pixel density and (**B**) fold changes of markers that are significantly different in cells treated with variety GN1 compared to cells treated with LPS. The experiment was performed at least in duplicate. Asterisks * represent statistically significant differences at *p* < 0.05, ** at *p* < 0.01 and ns means no significance.

Inflammation antibody microarray results are presented in Figure 8 showing that markers B-lymphocyte chemoattractant (BLC), fractalkine (TNFSF8), granulocyte–macrophage colony-stimulating factor (GM-CSF), IL-1α, IL-6, IL-12-p70, IL-13, lymphotactin (XCL1), monocyte chemoattractant protein (MCP)-1, macrophage colony-stimulating factor (M-CSF), monokine induced by gamma interferon (MIG), stromal cell-derived factor (SDF)-1, C-C motif ligand (CCL) 1, and TNF-α were expressed in cells treated with LPS and SCD of variety GN1 (Figure 8A). However, among those markers, the expression of markers GM-CSF and IL-6 were significantly decreased in cells treated with GN1 compared to the control LPS, while expression of markers TNFSF8 and CCL1 was increased (Figure 8B). Table 1 presents the inflammation antibody microarray results showing the fold change of proteins in cells treated with GN1 compared to control LPS.

In summary, the biochemical results suggest that different varieties of soybean had different protein profiles and concentrations, and SCD could inhibit the enzymatic activity of 5-LOX, COX-2, and iNOS biochemically. In vitro analysis demonstrated that the treatment with SCD could decrease the production of IL-1β and IL-6 in macrophages compared to the control LPS. Western blot results showed no statistically different expression of marker COX-2, and decreased expression in markers p65, p50, and iNOS. No significant difference was found in the production of TNF-α compared to the control LPS. Correlation analysis found that higher β-conglycinin content negatively correlated with production of inflammatory markers such as TNF-α and nitrite, while protein concentration was not found to have any strong correlation with the production of inflammatory markers. Inflammatory microarray results found decreased expression of GM-CSF and IL-6 in cells treated with SCD variety GN1 compared to LPS.

## 3. Discussion

Soybean and soybean bioactive compounds have been established to exhibit anti-inflammatory properties [18]. Previous studies have shown that soybean-derived lunasin could ameliorate inflammation through inhibition of p50 and p65 translocation that is needed for the activation of the NF-κB signaling pathway and reducing intestinal oxidative stress [19,20]. Additionally, higher lunasin concentration in dietary supplements was associated with higher antioxidant capacity [21]. Glycinin and β-conglycinin make up most of the protein content of soybean, and β-conglycinin in particular had been shown to be able to reduce obesity and LDL cholesterol, as well as inhibiting lipid accumulation and lowering inflammation [4,22,23,24]. Peptides from soybean were also found to be able to reduce intestinal inflammation through maintenance of intestinal mucosal integrity and suppression pro-inflammatory biomarkers [25,26,27]. Peptides from β-conglycinin and glycinin-rich fraction had also been found to inhibit colon cancer proliferation and inflammation [28].

Our current study simulated a complete digestion that mimicked as closely as possible actual digestion happening in the human body. We used various simulated digestion fluids and enzymes pronase E and viscozyme in addition to pepsin and pancreatin [29,30]. These enzymes are active in the colon—pronase E in the proximal colonic tract, viscozyme in the distal colonic tract—as part of colonic and full digestion, respectively. Research on colonic digestion has been mainly focused on carbohydrates. However, digestion of protein in the colon is important to observe the effect of its metabolites on gut and colonic health [31,32,33].

Gel electrophoresis analysis showed protein profile of different varieties of soybean and further densitometry analysis allowed us to quantify the percentage of each protein. The glycinin-null varieties (GN1 and GN3) were found to have the lowest glycinin content as expected, but also the highest β-conglycinin content. These varieties had the highest degree of hydrolysis, suggesting its higher digestibility. This result was in line with our previous study that showed higher glycinin content was correlated with lower degree of hydrolysis [34]. Gel electrophoresis analysis results also showed the presence of Kunitz trypsin inhibitor and Bowman–Birk inhibitors, both of which had been evaluated to have anti-inflammatory potential [35].

A biochemical study of soybean digests from the three phases of digestion suggested that soybean colonic and full digests responded better to the treatments, as shown by higher COX-2 inhibition. This was possibly due to enzymes in the colon releasing more bioactive peptides that were not hydrolyzed in the small intestine [36]. Further biochemical analysis showed that SCD were promising inhibitors of COX-2. However, they were not selective inhibitors of COX-2 as they also inhibited COX-1. The COX enzyme has been shown to have two different isoforms: COX-1, which was constitutively expressed in cells, and COX-2, which was induced during the event of inflammation. Selective inhibitors of COX-2 were developed based on this and in hope of also lessening the gastrointestinal toxicity which resulted from COX-1 inhibition [37,38]. However, a recent study showed that COX-1 inhibition might contribute to a decreased risk of colorectal cancer [39]. Therefore, there could be more potential for the health benefits of soybean colonic digests.

SCD was also shown to have the potential to inhibit the enzymatic activities of 5-LOX and iNOS. Varieties 88, GN3, and 93 were shown to have the highest inhibitory activity for enzymes COX-2, 5-LOX, and iNOS, respectively. Additionally, we found that the inhibition of COX-2 and 5-LOX enzymatic activity positively correlated with β-conglycinin content, indicating that higher β-conglycinin content could potentially indicate higher anti-inflammatory effects. β-Conglycinin is the vicilin storage protein of soybean, and it was evaluated to have multiple health benefits including modulating lipid accumulation and reducing risk of obesity and nonalcoholic fatty liver disease as reviewed by [40]. Vicilin is also present in other legumes such as mung bean, cowpea, and adzuki bean with increasing interest in its potential health benefits [41,42,43]. One study [42] found that β-vignin, a vicilin-like protein from adzuki bean, was able to reduce the activation of IL-8 to basal level [42]. Therefore, β-conglycinin from the SCD samples potentially contributed to anti-inflammation through the inhibition of the NF-κB signaling pathway and inhibition of inflammation-related enzymes as was shown in the correlations.

Cell viability assay was performed to test the potential cytotoxic effect of SCD on RAW 264.7 macrophages, and our results suggested that SCD did not have any cytotoxic effect. This result agreed with other studies that reported no cytotoxicity of soybean [44,45]. In vitro studies also found that soybean bioactive compounds could decrease the levels of pro-inflammatory markers such as TNF-α, IL-6, IL-1β, and nitric oxide production [46,47], which mostly agreed with our results, except that our findings did not find a significant reduction in TNF-α. Studies conducted on different legumes also found potential anti-inflammatory activities that were exerted through the downregulation of these markers such as in selenium-enriched black soybean, green pea, sword bean, and velvet bean [48,49,50,51]. Therefore, our tested SCD had similar anti-inflammatory potential as other legumes.

Western blot results showed that the expression of other pro-inflammatory markers tested such as iNOS, p65, and p50, was decreased in comparison to the control LPS. The subunits p65 and p50 are important components of the NF-κB signaling pathway since their nuclear translocation are needed to activate the pathway [52]. In addition, the results also showed a slight increase in the expression of IκB-α compared to the untreated control but short of significance (*p* > 0.05). This marker has been found to be anti-inflammatory by inhibiting the transcription factor of NF-κB by preventing it to bind with DNA [53,54]. However, more studies are needed to confirm the inhibition of subunits p65 and p50 translocation from cytosol to nucleus. We were limited in our observation of this due to our study using whole-cell extracts. Studies have shown that cells treated with soybean and soybean bioactive compounds decreased expression of iNOS and p65 [55,56]. Thus, this suggests that SCD could potentially inhibit NF-κB activation and iNOS enzyme expression at protein level.

Markers GM-CSF and IL-6 in cells treated with SCD variety GN1 were significantly decreased compared to LPS. Variety GN1 was the variety with highest β-conglycinin content and had been found to perform best in lowering the pro-inflammatory biomarkers. Thus, it was selected for the inflammation microarray. IL-6 is a pro-inflammatory cytokine that is important for both immune response and the progression of chronic inflammation, as well as being implicated in the progression of several diseases [57,58,59,60]. Both of our results from inflammation microarray and ELISA demonstrated that SCD treatment could inhibit the production and expression of IL-6. GM-CSF is a cytokine that is involved in immune response and inflammation cascade, activating the adaptive immune response while also being implicated in the activation of the NF-κB signaling pathway and increases the mRNA expression of pro-inflammatory biomarkers [61,62]. A recent study also found that GM-CSF is a regulator of macrophage proliferation, inducing differentiation into pro-inflammatory M1 macrophages, which is important due to its anti-bacterial property in immune response but also suppressed its wound healing function [63]. Thus, the reduction in GM-CSF expression by SCD could lead to anti-inflammatory potential of inhibiting the NF-κB signaling pathway and reducing production of inflammatory biomarkers.

Additionally, we found a significant increase in expression of markers fractalkine or TNFSF8 and CCL1 in cells treated with SCD variety GN1 compared to the control LPS. Fractalkine is a chemokine that binds to its receptor on the surface of macrophages and functions as a modulator of macrophage proliferation and differentiation into pro-inflammatory M1, as well as production of pro-inflammatory cytokines [64,65]. CCL1 is a chemokine that functions as chemoattractant of leukocytes to inflammation sites and has been implicated in several diseases, making it a potential therapeutic target for inflammation-related diseases [66,67,68,69]. However, a recent study also observed that CCL1 as a ligand of chemokine receptor CCR8 could have protective effect against intestinal colitis [70]. This could have implications in the potential prevention of other diseases such as inflammatory bowel disease (Crohn’s disease and ulcerative colitis), cancer, and other inflammation-related diseases.

**Figure 9:**
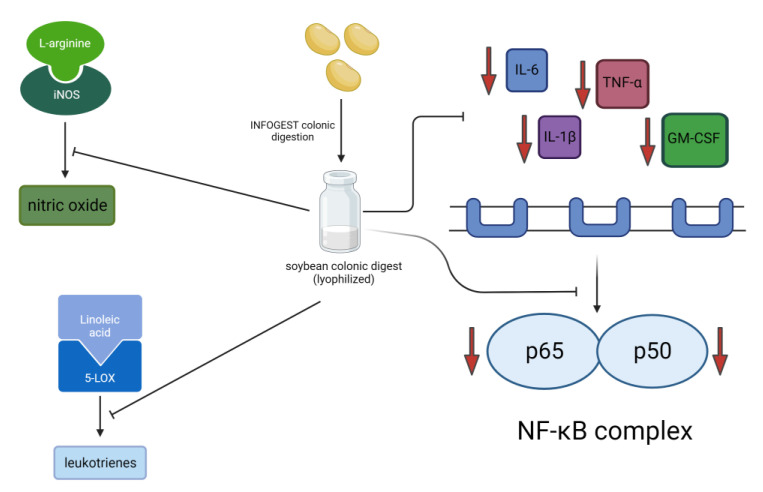
Diagram indicating soybean digests ameliorated inflammation through modulation of the NF-κB pathway and inhibited the activity of pro-inflammatory enzymes. Treatment with soybean digests inhibited the activity of enzymes 5-lipoxygenase and inducible nitric oxide and lowered the expression of subunits p65 and p50, and pro-inflammatory cytokine production. Downward red arrows mean inhibition or decrease of the particular marker.

In summary, soybean varieties with the lowest glycinin and highest β-conglycinin content were found to be the most digestible. We showed that β-conglycinin content in SCD correlated with higher anti-inflammatory effect as it was associated with higher biochemical inhibition of COX-2 and 5-LOX, and lower production of TNF-α and nitrites. We also showed that treatment with SCD significantly lowered the expression of iNOS, thus indicating the anti-inflammatory exerted through both the reduced expression and enzymatic activity of iNOS that led to lower nitric oxide production. SCD also significantly reduced the levels of IL-6 and expression of p65 and p50 markers (Figure 9). Differences in the soybean protein composition might be of practical significance for variety selection to fit their specific food and health applications. Soybean anti-inflammatory capacity was associated with different protein profiles and peptides embedded and released during gastrointestinal digestion.

## 4. Materials and Methods

### 4.1. Materials

Seventeen soybean varieties and two additional glycinin-null (GN1 and GN3) varieties were provided by Benson Hill (St. Louis, MO, USA) and stored at 4 °C. Seven representative soybean colonic digests [34] were selected for screening to determine anti-inflammatory indicators [34]. A DC protein assay kit, 2× Laemmli sample buffer, 10× Tris/glycine/SDS buffers, mini-PROTEAN^®^ TGX™ gels (4–20%, 15 well-comb, 15 μL), and Precision Plus Protein™ Dual Xtra standard were purchased from Bio-Rad (Hercules, CA, USA). Simply Blue Safe Stain was purchased from Invitrogen (Carlsbad, CA, USA). A COX Fluorescent Inhibitor Screening assay kit was purchased from Cayman Chemical (Ann Arbor, MI, USA). An inducible nitric oxide synthase inhibitor assay kit (Fluorometric) was purchased from Abcam (Boston, MA, USA). Cell line RAW 264.7 was purchased from the American Type Culture Collection (ATCC^®^ TIB–71, Manassas, VA, USA). CellTiter 96^®^ Aqueous One Solution was purchased from Promega Corporation (Madison, WI, USA). The ELISA kits IL-1β and IL-6 were purchased from RayBiotech Inc. (Norcross, GA, USA). The antibodies anti-p50 (epitope 580–598, polyclonal), anti-p65 (epitope 1–286, monoclonal), anti-COX-2 (epitope 580–598, monoclonal), anti-IκB-α (epitope 1–317, monoclonal), β-tubulin (epitope 210–444, monoclonal), and anti-iNOS (epitope 1126–1144, polyclonal) were purchased from Invitrogen (Carlsbad, CA, USA). The antibody GAPDH (epitope 1–335, polyclonal) was purchased from Santa Cruz (Santa Cruz, CA, USA). Secondary anti-rabbit and anti-mouse IgG Horseradish peroxidase were purchased from Cytiva (Marlborough, MA, USA). Mouse inflammation antibody array C1 was purchased from RayBiotech (AAM-INF-1-4, Norcross, GA, USA). All other reagents were purchased from Sigma-Aldrich (St. Louis, MO, USA) unless stated otherwise.

### 4.2. Methods

#### 4.2.1. Preparation of Defatted Soybean Flour

Soybean seeds were weighed, ground using a coffee grinder, and passed through a 1.16-mm sieve to obtain homogenous particle-size materials. The soybean flour was stored at −20 °C until used. It was then defatted according to the methods described by de Mejia et al. (2004) [71] with some modifications using the Soxhlet extraction system. Hexane (ACS standard) was used as the solvent of choice, and the system was let run for 5 h. The defatted flour was left under the hood to air-dry overnight before being stored at 4 °C.

#### 4.2.2. Protein Extraction and Quantification

Protein was extracted from the defatted samples using the method described in [71] with a minor modification. Briefly, 0.075 g of the defatted flour was placed in 1.5 mL of extracting buffer (0.05 M Tris-HCl buffer, pH 8.2) in an Eppendorf tube. Mixtures were vortexed and placed in an ultrasonic bath (Bransonic model 2510, Branson Ultrasonic Corporation, Danbury, CT, USA) for 70 min, and vortexed periodically every 10 min to avoid settlement. The protein concentration of the soybean samples was evaluated using the DC protein assay according to the manufacturer’s protocol.

#### 4.2.3. Simulated Gastrointestinal Digestion

The simulated gastrointestinal digestion process was performed according to [34]. The enzymatic activity assays of pepsin and trypsin contained in pancreatin were assessed using the methods described in the supplementary sections of the INFOGEST material provided by [29]. The digestion process was divided into five phases: oral, gastric, duodenal, colonic, and full. However, only the resulting digests from the duodenal, colonic, and full digestion phases were tested. Preliminary results suggested colonic digests responded better to treatments; therefore, it was the condition that was used for all soybean varieties. The soybean digests were centrifuged at 4 °C and 4000 rpm for 40 min after stopping the digestion process by heating. The supernatant was freeze-dried and stored at −80 °C until further use.

#### 4.2.4. Peptide Identification

Peptides (2 mg/mL) were prepared for analysis by LC-QTOF-MS/MS with an Alliance 2795 HPLC system coupled to an Ultima mass spectrometer on positive-ion electrospray mode (Water, Milford, MA, USA). Gradient mobile phase A (95% water, 5% acetonitrile, 0.01% formic acid), gradient mobile phase B (95% acetonitrile, 5% water, 0.1% formic acid), a flow rate of 400 μL/min, and a signal of 280 nm for PDA detector recording were used as parameters. The resulting mass spectra were analyzed using MassLynx V4.1 (Waters Corp, Milford, MA, USA). The parent protein of peptides analyzed was obtained by comparing the sequences in the BLAST database (https://blast.ncbi.nlm.nih.gov/Blast.cgi; accessed on 14 September 2022). The BIOPEP database (https://biochemia.uwm.edu.pl/en/biopep-uwm-2/; accessed on 14 September 2022) was accessed to obtain the potential bioactivity. PepDraw (http://www.tulane.edu/~biochem/WW/PepDraw/; accessed on 14 September 2022) was used to analyze the mass, isoelectric point (pI), net charge, and hydrophobicity.

#### 4.2.5. SDS–PAGE Electrophoresis

SDS–PAGE was used to analyze the protein profiles of the samples. Briefly, protein (20 μg) was loaded into each gel well. Electrophoresis was performed at 200 V, 400 mA for 35 min using Tris/Glycine/SDS. The gel was washed three times with distilled water, stained with SimplyBlue stain at room temperature for 1 h, and destained with distilled water. The image was captured using colorimetric imaging by ImageQuant 800 Fluor system. The images were further analyzed using the ImageJ software to determine the proportion of glycinin and β-conglycinin. These proportions were presented as percentages of the total protein content.

#### 4.2.6. Degree of Hydrolysis

The determination of the degree of hydrolysis of each sample was performed according to the method described by [72] with some modifications. Briefly, O-phtaldialdehyde (OPA) reagent was prepared by dissolving 10 mg of phtaldialdehyde in 250 μL of ethanol, 20 μL of 2-mercaptoethanol, and 9.8 mL phosphate-buffered saline (PBS). Serine (0–0.2 mg/mL) was used to create a standard curve. Water (140 μL) and OPA reagent (100 μL) were added to 10 μL of the sample (1 mg/mL). The reaction was read at 340 nm excitation and 440 nm emission. The degree of hydrolysis was determined using the following Equations (1)–(3):

(1) $$\mathrm{Degree}\;\mathrm{of}\;\mathrm{hydrolysis}\;\left(\mathrm{DH}\right)=\frac{h}{h_{\mathrm{hot}}}\times 100$$

(2) $$h=\frac{\frac{\mathrm{Serine}-\mathrm{NH}2-\beta }{\alpha }}{g\;\mathrm{of}\;\mathrm{protein}}$$

(3) $$\mathrm{Serine}-\mathrm{NH}2=\frac{\mathrm{ODsample}-\mathrm{ODblank}}{\mathrm{ODs}\tan\mathrm{dard}-\mathrm{ODblank}}\times 0.9516\frac{\mathrm{meqv}}{L}\times 0.1\times \frac{100}{X\times P}$$

where h_hot_ = 7.8, α = 0.970, and β = 0.342. These values were reported by [73] specifically for soy samples.

#### 4.2.7. Cyclooxygenase (COX)-1 and -2 Inhibitor Screening Fluorometric Biochemical Assay

The cyclooxygenase (COX, E.C.1.14.99.1) -1 and -2 inhibitor screening assay was performed according to the manufacturer’s protocol with some modifications. DuP-697 (60 μM) was chosen as the positive control for COX-2, while SC-560 (66 μM) was selected as the positive control for COX-1. Both were included with the assay kit. Blank digest (digestion run without soy flour) was used as the negative control. The blank digest was calculated to comprise 39% of the samples, and the results were scaled accordingly. COX-2 assay was initially run with only seven representative digests obtained from three digestion conditions (duodenal, colonic, and full). It was decided to evaluate the colonic digests. Each well contained 1 mg/mL soluble soybean digest. The percentage inhibition was calculated using the following Equation (4):

(4) $$\%\;\mathrm{Inhibition}=\frac{\mathrm{initial}\;\mathrm{activity}\;-\;\mathrm{sample}\;\mathrm{activity}}{\mathrm{initial}\;\mathrm{activity}}\times 100$$

#### 4.2.8. 5-Lipoxygenase (LOX) Inhibitory Screening Biochemical Assay

5-Lipoxygenase (EC 1.13. 11.34) inhibition assay was performed according to the protocol described in [74]. The blank digest was chosen as the negative control. Trolox (0–50 μM) was used as a standard curve. Zileuton (100 μM) was used as a positive control. Each well contained 300 μL of reaction solution consisting of approximately 1 mg/mL soluble soybean digest content, 4.5 μM fluorescein, 100 mM Na-borate buffer (pH 9), 200 μM linoleate solution, and 0.5 EU soybean LOX. The following Equation (5) was used to calculate the percentage of enzyme activity inhibition:

(5) $$\%\;\mathrm{inhibition}=\left(1\;-\;\frac{V_{a}}{V_{c}}\right)\;\times \;100$$

where V_a_ = decreased rate of sample, and V_c_ = decreased rate of the control, calculated from the highest slope of the curve. Inhibition was also presented as μM Trolox equivalent.

#### 4.2.9. Inducible Nitric Oxide Synthase (iNOS) Screening Biochemical Assay

The iNOS (EC 1.14.13.39) inhibition assay was performed according to the protocol from the manufacturer. Blank digest served as a negative control, while diphenyleneiodonium chloride (DPI, 1 mM) served as a positive control. Each well contained a sample with 0.5 mg/mL soluble protein content. Inhibition of iNOS was calculated using the following Equation (6):

(6) $$\%\;\mathrm{inhibition}=\frac{\mathrm{Absorbance}\;\mathrm{of}\;\mathrm{sample}}{\mathrm{Absorbance}\;\mathrm{of}\;\mathrm{blank}}\times 100$$

#### 4.2.10. Cell Culture and Cell Viability Assay

The macrophage cell line RAW 264.7 was cultured in Dulbecco’s modified Eagle medium (DMEM), 1% penicillin/streptomycin, 1% sodium pyruvate, and 10% FBS at 37 °C in 5% CO_2_/95% air. Cells were seeded in 96-well plates at a confluence of 6 × 10^4^ cells/well and treated with soybean digests (0.1, 0.25, 0.5, 1, and 2.5 mg/mL) for 30 min, then stimulated with LPS (1 μg/mL) and incubated for a total of 24 h. Dexamethasone (20 μM) was used as the positive control. Cell viability was measured using MTS, composed of tetrazolium salt that is reduced by dehydrogenase enzyme in cells to formazan, a blue-colored product whose intensity is related to the number of living cells. The percentage of cell viability was calculated using Equation (7):

(7) $$\%\;\mathrm{Cell}\;\mathrm{Viability}=\frac{\mathrm{Absorbance}\;\mathrm{of}\;\mathrm{treatment}\;}{\mathrm{Absorbance}\;\mathrm{of}\;\mathrm{control}}\times 100$$

#### 4.2.11. Measurement of Nitrite Production in Cell Supernatant

The measurement of nitrite production was performed according to the protocols described in [22]. The Griess reaction was used for determining nitrite production by mixing 100 μL of cell supernatant and 100 μL of Griess reagent in a 96-well plate. The plate was incubated for 5 min in the dark at room temperature before absorbance was measured at 550 nm. Nitrite production was calculated using a sodium nitrite (0–200 μΜ) standard curve. IC_50_ (half-maximal inhibitory concentration) value was calculated to indicate the potency of the SCD, with lower IC_50_ value indicating higher potency.

#### 4.2.12. Measurement of IL-1β, IL-6, and TNF-α Levels in Cell Supernatant

RAW 264.7 macrophage cells were seeded in a 6-well plate at a confluency of 1.7 × 10^6^ cells/well. The cells were stimulated with 1 μg/mL LPS and treated with the IC_50_ values of nitrite production of selected soybean digests based on the biochemical results (Var 81, IC_50_ = 1.13 mg/mL; Var 87, IC_40_ = 2.4 mg/mL; Var 96, IC_50_ = 1.49 mg/mL; Var 103, IC_50_ = 1.18 mg/mL; Var 108, IC_50_ = 1.43 mg/mL; Var GN1, IC_50_ = 1.20 mg/mL; Var GN3, IC_50_ = 1.46 mg/mL). The levels of IL-1β, IL-6, and TNF-α produced were determined by enzyme-linked immunoabsorbent assay (ELISA) assay kits performed according to the manufacturer’s protocols. Levels of IL-1β, IL-6, and TNF-α were analyzed using the sandwich ELISA method.

#### 4.2.13. Western Blot from Cell Lysates

Western blot was used to measure the expressions of markers p65, p50, COX-2, iNOS, and IκB-α. Briefly, the protein from cell lysates (20 μg) that has previously been separated by electrophoresis was transferred to PVDF membranes (Cytiva, Marlborough, MA, USA) and then blocked with 5% (*w*/*v*) nonfat dry milk, incubated overnight at 4 °C with primary rabbit antibodies (1:1000 *v*/*v*) for subunits p65 and p50, COX-2, GAPDH, IκB-α, and iNOS, washed, and incubated with secondary anti-rabbit IgG Horseradish peroxidase-linked (Cytiva, Marlborough, MA, USA) antibodies (1:2000 *v*/*v*). Clarity Western ECL substrate (Bio-Rad, Hercules, CA, USA) was applied to visualize the protein bands using the ImageQuant 800 system. Densitometry analysis of the resulting images was performed using the ImageJ software, and results were presented as fold-change comparison to the control LPS normalized to GAPDH.

#### 4.2.14. Inflammation Antibody Array in Cell Supernatant

Measurement of inflammation-related markers was performed using the mouse inflammation antibody array C1 according to the manufacturer’s protocol (RayBiotech, Peachtree Corners, GA, USA). The intensity of the proteins expressed was analyzed using the ImageJ software.

#### 4.2.15. Statistical Analysis

Data were expressed as means of two or three independent replicates. The results between samples were compared by one-way and two-way analysis of variance (ANOVA) followed by Tukey’s test for multiple comparisons. Student’s *t*-test was used to compare the results between treatments in inflammation microarray. Differences were considered statistically significant at *p* < 0.05. Pearson correlation coefficient was used to determine the correlation of results; a correlation was considered at r > 0.5. GraphPad Prism 9 (GraphPad Software Inc., San Diego, CA, USA) was used to perform all the statistical analyses.

## 5. Conclusions

Higher β-conglycinin content correlated with higher anti-inflammatory potential, as it was associated with higher inhibition of inflammatory-related enzymes and lowered production of pro-inflammatory biomarkers. Soybean digests inhibited the enzymatic activity of 5-LOX and iNOS. Inflammatory microarray results showed significant decrease in the expression of markers GM-CSF and IL-6 in cells treated with GN1 digest compared to LPS. SCD has the potential to inhibit the NF-κB inflammation signaling pathway through decreasing the expression of p65 and p50 subunits, thus inhibiting nuclear translocation that is needed for the activation.

## Figures and Tables

**Table 1:** Inflammation antibody microarray results show the fold change of markers in cells treated with GN1 compared to control LPS.

Marker	Abbreviation	Role	Fold Change Relative to Control LPS	
			LPS	GN1
Granulocyte–macrophage colony-stimulating factor	GM-CSF	Stimulating the production of white blood cells including granulocytes and macrophages	1.00 ± 0.02 ^a^	0.01 ± 0.05 ^b^
Interleukin-6	IL-6	Pro-inflammatory cytokine; stimulates synthesis of acute phase proteins (i.e., CRP) and serum amyloid A; inhibits albumin production	1.00 ± 0.03 ^a^	0.45 ± 0.03 ^b^
Fractalkine	TNFSF8	Migration, adhesion, and proliferation of multiple types of cells including T-cells	1.00 ± 0.03 ^a^	1.42 ± 0.03 ^b^
C-C motif ligand 1	I-309 (TCA-3/CCL1)	Attracts macrophages to the inflammation site	1.00 ± 0.07 ^a^	1.80 ± 0.07 ^b^

Markers from inflammation microarray that showed significant difference (*p* < 0.05) in treatment with variety GN1 digest compared to control LPS. Fold change was calculated relative to control LPS with fold change lower than 1 indicating decrease in expression and higher than 1 indicating increase in expression. Different letters in each marker represent significant differences at *p* < 0.05.

## Data Availability

Data is contained within the article or Supplementary Materials.

## Supplementary Materials

The following supporting information can be downloaded at: https://www.mdpi.com/article/10.3390/ijms241512396/s1.

## Abbreviations

CCL, C-C Motif Chemokine Ligand; COX, Cyclooxygenase; GBR, Glycinin:β-Conglycinin Ratio; GM-CSF, Granulocyte–Macrophage Colony-Stimulating Factor; IL, Interleukin; INOS, Inducible Nitric Oxide Synthase; JAK/STAT, Janus Kinase-Signal Transducer and Activator of Transcription; LOX, Lipoxygenase; MAPK, Mitogen-Activated Protein Kinase; MCP, Monocyte Chemoattractant Protein; M-CSF, Macrophage Colony-Stimulating Factor; MIG, Monokine Induced by Interferon Gamma; NO, Nitric Oxide; NF-κB, Nuclear Factor kappa B; SCD, Soybean Colonic Digest; SDF-1, Stromal Cell-Derived Factor 1; TNF-α, Tumor Necrosis Factor-alpha.
