# Supplementary material for: Soybean (Glycine max) INFOGEST Colonic Digests Attenuated Inflammatory Responses Based on Protein Profiles of Different Varieties
Source: Int J Mol Sci. 2023 Aug 3;24(15):12396. doi: 10.3390/ijms241512396 (PMC10418973; doi:10.3390/ijms241512396)
Supplement: Supplementary file 1 [file ijms-24-12396-s001.zip › ijms-2514604-Supplemental Figures and Tables.pdf]

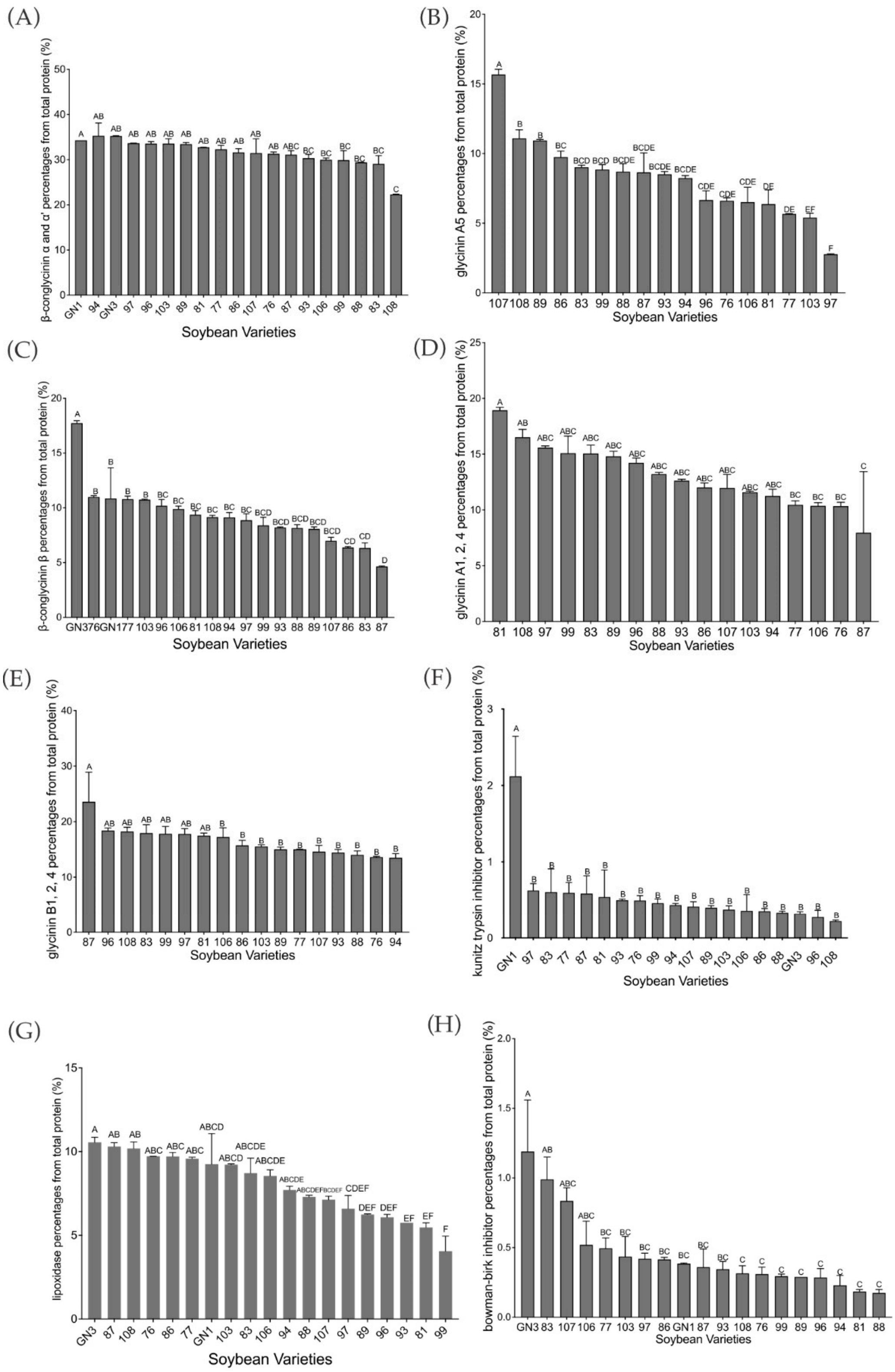

Supplemental Figure S1. The proportion of each protein content expressed as a percentage of total protein content (A) β-conglycinin subunits α and α' (B) Glycinin A5 (C) β-conglycinin β (D) Glycinin A1, 2, 4 (E) Glycinin B1, 2, 4 (F) Kunitz Trypsin Inhibitor (G) LOX (H) Bowman-Birk Inhibitor. Results are arranged from highest to lowest. Bars with different letters represent statistically significant differences according to one-way ANOVA and Tukey's multiple comparison tests ($p < 0.05$).

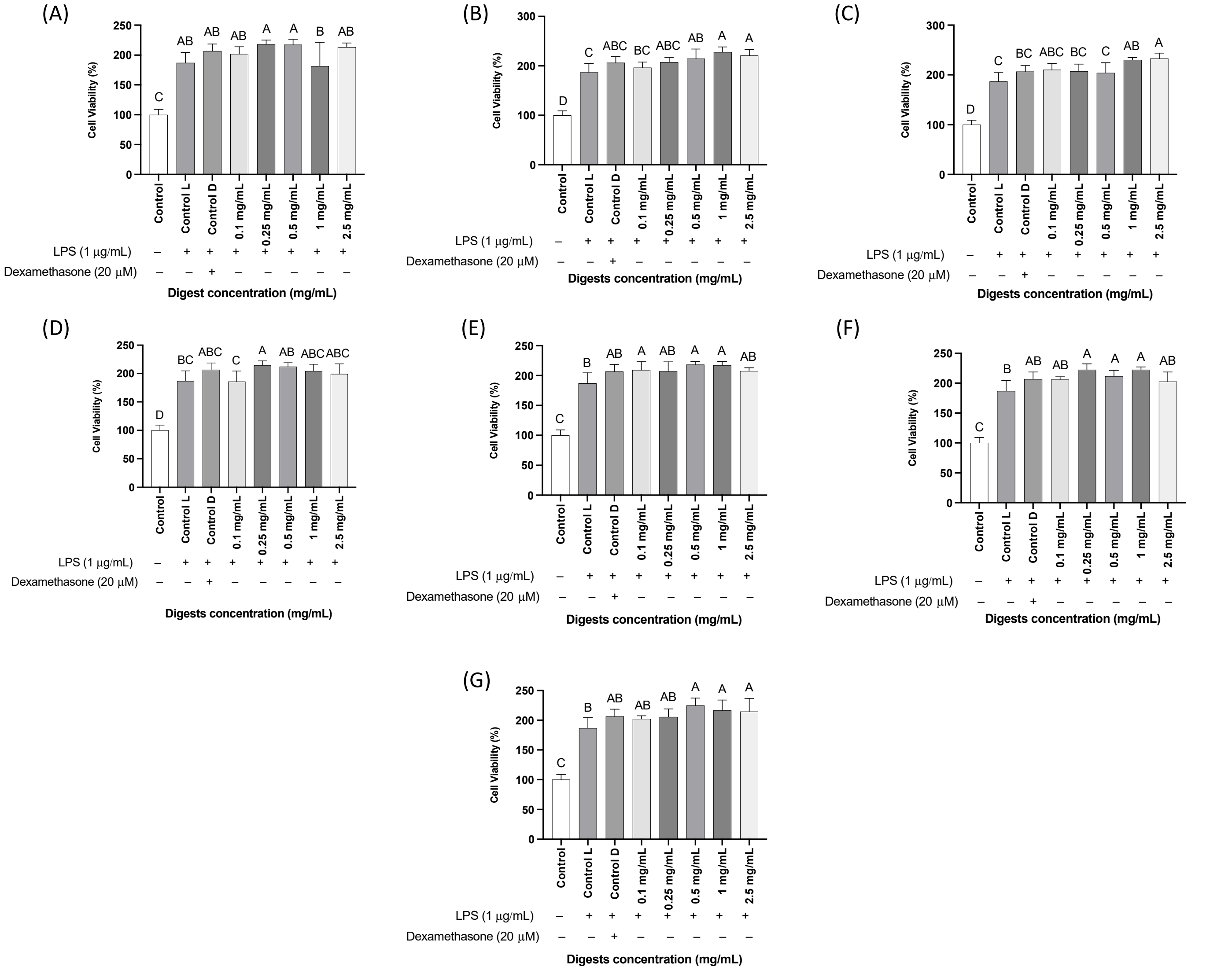

Supplemental Figure S2. Cell viability of different digests concentrations for the varieties (A) 81, (B) 87, (C) 96, (D) 103, (E) 108, (F) GN1 and (G) GN3. NS = no significance $p > 0.05$. The assay was done at least in duplicates. Data were analyzed using ANOVA (one-way) and Tukey's post hoc test. Bars with different letters represent statistically significant differences ($p < 0.05$)

Supplementary Table S1. Identification of proteins in soybean varieties and their respective approximate molecular weight ranges.

Protein	Molecular Weight (kDa)
**Lipoxygenase**	90 – 95
**β-conglycinin α'**	57 – 72
**β-conglycinin α**	57 – 68
**β-amylase**	61.5 – 63
**β-conglycinin β**	45 – 52
**Glycinin acidic subunit**	10 – 15, 30 – 38, 42 – 44
**Kunitz trypsin inhibitor (KTI)**	21.5 – 22.5
**Glycinin basic subunit**	18 – 20
**Bowman-Birk inhibitor (BBI)**	8 – 10
